# The Role of *Candida albicans* Secreted Polysaccharides in Augmenting *Streptococcus mutans* Adherence and Mixed Biofilm Formation: *In vitro* and *in vivo* Studies

**DOI:** 10.3389/fmicb.2020.00307

**Published:** 2020-02-28

**Authors:** Zaid H. Khoury, Taissa Vila, Taanya R. Puthran, Ahmed S. Sultan, Daniel Montelongo-Jauregui, Mary Anne S. Melo, Mary Ann Jabra-Rizk

**Affiliations:** ^1^Department of Oncology and Diagnostic Sciences, University of Maryland School of Dentistry, Baltimore, MD, United States; ^2^Ph.D. Program in Biomedical Sciences, University of Maryland School of Dentistry, Baltimore, MD, United States; ^3^Division of Operative Dentistry, Department of General Dentistry, University of Maryland School of Dentistry, Baltimore, MD, United States; ^4^Department of Microbiology and Immunology, University of Maryland School of Medicine, Baltimore, MD, United States

**Keywords:** *Candida albicans*, polysaccharide, matrix, *Streptococcus mutans*, mixed-biofilms, dental caries, fungal-bacteria interactions

## Abstract

The oral cavity is a complex environment harboring diverse microbial species that often co-exist within biofilms formed on oral surfaces. Within a biofilm, inter-species interactions can be synergistic in that the presence of one organism generates a niche for another enhancing colonization. Among these species are the opportunistic fungal pathogen *Candida albicans* and the bacterial species *Streptococcus mutans*, the etiologic agents of oral candidiasis and dental caries, respectively. Recent studies have reported enhanced prevalence of *C. albicans* in children with caries indicating potential clinical implications for this fungal-bacterial interaction. In this study, we aimed to specifically elucidate the role of *C. albicans*-derived polysaccharide biofilm matrix components in augmenting *S. mutans* colonization and mixed biofilm formation. Comparative evaluations of single and mixed species biofilms demonstrated significantly enhanced *S. mutans* retention in mixed biofilms with *C. albicans.* Further, *S. mutans* single species biofilms were enhanced upon exogenous supplementation with purified matrix material derived from *C. albicans* biofilms. Similarly, growth in *C. albicans* cell-free spent biofilm culture media enhanced *S. mutans* single species biofilm formation, however, the observed increase in *S. mutans* biofilms was significantly affected upon enzymatic digestion of polysaccharides in spent media, identifying *C. albicans* secreted polysaccharides as a key factor in mediating mixed biofilm formation. The enhanced *S. mutans* biofilms mediated by the various *C. albicans* effectors was also demonstrated using confocal laser scanning microscopy. Importantly, a clinically relevant mouse model of oral co-infection was adapted to demonstrate the *C. albicans*-mediated enhanced *S. mutans* colonization in a host. Analyses of harvested tissue and scanning electron microscopy demonstrated significantly higher *S. mutans* retention on teeth and tongues of co-infected mice compared to mice infected only with *S. mutans*. Collectively, the findings from this study strongly indicate that the secretion of polysacharides from *C. albicans* in the oral environment may impact the development of S. mutans biofilms, ultimately increasing dental caries and, therefore, *Candida* oral colonization should be considered as a factor in evaluating the risk of caries.

## Summary

In the mouth, a multitude of microbial species co-exist adhering to various oral surfaces. Within these microbial communities known as biofilms, complex inter-species interactions take place that can be synergistic in that the presence of one organism can enhance the colonization of another. Most notable among the microorganisms that reside in the oral cavity are the fungal species *Candida albicans* and the bacterial species *Streptococcus mutans*, the causative agents of oral candidiasis and dental caries, respectively. Interestingly, recent studies have reported enhanced prevalence of *C. albicans* in children with dental caries indicating a potential clinical implication for this fungal-bacterial interaction in the oral cavity. By taking different experimental approaches, our results provided new mechanistic insights into this complex interaction; specifically, we demonstrated a crucial role for the *C. albicans* derived secreted polysaccharides in enhancing *S. mutans* retention and proliferation within a biofilm. Importantly, a mouse model of co-infection was developed to demonstrate that the prevalence of *C. albicans* in the oral cavity significantly increases *S. mutans* colonization on teeth and oral tissue. Combined, the generated findings indicate a potential role for *C. albicans* in increasing the risk for the development of dental caries *via* its interaction with *S. mutans*.

## Introduction

The oral microbiome is one of the most complex environments harboring diverse microbiota that co-exist in equilibrium (Wade, 2013; Krom et al., 2014; Xu and Dongari-Bagtzoglou, 2015; Sultan et al., 2018). This equilibrium is crucial for maintaining oral health as an imbalance potentiates the dominance of pathogenic species, which may lead to the development of disease (Jenkinson and Lamont, 2005; Duran-Pinedo and Frias-Lopez, 2015; Bertolini et al., 2019). In the oral cavity, microorganisms exist within highly organized and structured microbial communities referred to as biofilms, where microbial cells are embedded within a self-produced extracellular polymeric substance (Jenkinson et al., 1990; Kolenbrander et al., 2002; Rickard et al., 2003; Vu et al., 2009). In this environment, extensive inter-species interactions take place, which can be synergistic in that the presence of one organism generates a niche for others, enhancing colonization and retention (Xiao et al., 2012; Sultan et al., 2018).

Dental plaque is one of the earliest biofilm models studied as it is composed of diverse microbial species co-adhering to the surface of teeth and interacting within a matrix of exopolysaccharides (Kidd and Fejerskow, 2004; Zero et al., 2009; Klein et al., 2015; Tanner et al., 2018; Valm, 2019). Within the plaque biofilm, the bacterial species *Streptococcus mutans* is considered to be the critical effector for the development of carious lesions (Klein et al., 2015). Dental caries (or tooth decay) is among the most prevalent human diseases characterized by localized and irreparable destruction of the tooth (Isalm et al., 2007; Zero et al., 2009; Rouabhia and Chmielewski, 2012). Combined with its strong binding to teeth, the ability of *S. mutans* to produce large quantities of glucans, produce acid and survive in acidic environment, ultimately results in dissolution of hydroxyapatite in tooth enamel and dentin (Isalm et al., 2007; Koo et al., 2013; Lemos et al., 2013; Klein et al., 2015; Valm, 2019). Interestingly, although *S. mutans* has long been considered the main cariogenic species, recent evidence seems to attribute a potential role for the fungal species *Candida albicans*, *via* interactions with *S. mutans* (Metwalli et al., 2013; Falsetta et al., 2014; Pereira et al., 2018; Xiao et al., 2018).

Similar to *S. mutans*, *C. albicans* is a natural commensal colonizer of the oral cavity (Calderone, 2012; Krom et al., 2014; Sultan et al., 2018). However, under conditions of immune suppression or changes in the host environment, this opportunistic organism can rapidly transition to a pathogen causing a variety of infections, most commonly oral candidiasis (Fidel, 2011; Williams and Lewis, 2011; Jabra-Rizk et al., 2016).

*Candida albicans* is a dimorphic species able to switch morphology between yeast and hyphal forms, a property that is central to its pathogenesis and ability to form biofilms (Jabra-Rizk et al., 2004; Ramage et al., 2005; Nett and Andes, 2006; Calderone, 2012; Wall et al., 2019). Microbial biofilms produce extracellular matrix that confers such properties as adherence and drug resistance. The *C. albicans* biofilm matrix is complex and largely composed of polysaccharides such as β-1,3-glucan, β-1,6-glucan, and mannans which form the mannan–glucan complex (MGCx) (Al-Fattani and Douglas, 2006; Nett et al., 2007; Nett et al., 2011; Mitchell et al., 2015). Comprehensive analysis of *C. albicans* extracellular matrix demonstrated that β-1,3 glucan is a relatively minor matrix component, whereas α-mannan and β-1,6 glucan constitute 85 and 14% of the matrix carbohydrate fraction, respectively. Using genetic and biochemical approaches to determine the contributions of these constituents to matrix function, a comprehensive study by Mitchell et al. (2015) indicated that matrix development entails coordinated delivery of the individual polysaccharides and that each of the components are required for matrix function.

The interactions between *C. albicans* and streptococci in the oral cavity are well-established and are considered to be synergistic in nature (Jenkinson et al., 2008; Diaz et al., 2012; Xu and Dongari-Bagtzoglou, 2015; Ellepola et al., 2017; Koo et al., 2018; Montelongo-Jauregui and Lopez-Ribot, 2018; Montelongo-Jauregui et al., 2019). In addition to physical associations, metabolic interactions have also been described where by utilizing lactic acid, streptococci provide *C. albicans* with a carbon source for growth, which, in turn, results in lower oxygen tension to levels advantageous to facultative streptococci (Jenkinson and Lamont, 2005). Moreover, the *S. mutans* exoenzymes glucosyltranferases (Gtfs) are known to be deposited onto the surface of *C. albicans* cells and, as a consequence of sucrose breakdown, produce glucans on the fungal cell wall which facilitate the adherence between the two microorganisms (Bowen and Koo, 2011; Falsetta et al., 2014; Koo and Bowen, 2014). Interestingly, *in vitro* studies investigating the cariogenic potential of *C. albicans* have been conflicting; a recent study using dentine slabs reported that *C. albicans* increases dentine demineralization by enhancing the cariogenic potential of *S. mutans* (Sampaio et al., 2019). In contrast, other studies indicated that *C. albicans* raises the pH of the environment and has low enamel demineralization potential (Hubertine et al., 2016; Eidt et al., 2019). These discrepancies are most likely due to different experimental conditions used and are not accurately reflective of the oral environment.

Significantly, several studies have reported high prevalence for *S. mutans* in dental biofilms where *C. albicans* resides, suggesting that this fungal-bacterial interaction may contribute to dental caries (Barbieri et al., 2007; Jarosz et al., 2009; Raja et al., 2010; Sztajer et al., 2014; Hwang et al., 2015; Hwang et al., 2017; Yang et al., 2018; Lobo et al., 2019). The potential of *C. albicans* to induce dental caries as a consequence of its distinct ability to produce and tolerate acids was supported by findings from a study by Klinke et al. (2011), where *C. albicans* was shown to be capable of causing caries in rats. Of more importance, however, are findings from clinical studies positively correlating the prevalence of *C. albicans* in the oral cavity with *S. mutans* prevalence, and occurrence of caries in children (de Carvalho et al., 2006; Raja et al., 2010; Fragkou et al., 2016). Furthermore, a recent systematic review and meta-analysis concluded that children younger than 6 years of age that harbor *C. albicans* in their oral cavity, have more than five times higher odds of developing early childhood caries (ECC) compared to children without *C. albicans* (Xiao et al., 2018). The combined findings from these animal and clinical studies are strongly suggestive of a potential cariogenic role for *C. albicans* in mediating ECC development, *via* synergistic physical and metabolic interactions with *S. mutans*.

Therefore, given the clinical implications of this fungal-bacterial association within oral biofilms, understanding the mechanisms of their interactions as they co-exist may aid in the design of targeted interventional therapeutic strategies for the prevention of dental caries. To that end, by taking different experimental approaches, we aimed to provide mechanistic insights into this synergistic interaction between *C. albicans* and *S. mutans* within a mixed biofilm. Specifically, experiments were designed to elucidate the role of the *C. albicans*-derived polysaccharide components of the extracellular fungal biofilm matrix in augmenting *S. mutans* colonization and retention. Importantly, an animal model of oral co-colonization was adapted to demonstrate this phenomenon in a host. Combined, the findings from this study warrant further investigations into potential therapeutic strategies targeting polymicrobial oral conditions such as dental caries.

## Experimental Procedures

### Strains and Growth Conditions

The *Streptococcus mutans* wild-type strain UA159 was used in all experiments, and a GFP-tagged *S. mutans* wild-type strain (UA159-GFP) (kindly provided by Dr. Bastiaan Krom) was used where indicated. *S. mutans* isolates were maintained on Brain Heart Infusion (BHI) (Sigma-Aldrich, United States) agar plates; for experiments, a few isolated colonies of *S. mutans* were suspended in BHI broth and incubated overnight in an anaerobic jar at 37°C with shaking. Cells were washed twice with PBS and cell density was adjusted to final concentration as indicated based on the optical density at 600 nm (OD_600_) measurements. The standard wild-type *C. albicans* SC5314 strain was used in all experiments (Gillum et al., 1984). *C. albicans* was maintained on yeast extract, bacto-peptone, and dextrose (YPD) agar plates; for experiments, a single *C. albicans* colony was suspended in YPD broth and grown overnight at 30°C with shaking. Cells were harvested and washed twice with PBS, and final cell density was adjusted as indicated based on OD_600_ measurements. For species isolation, the *Candida*-specific chromogenic media CHROMagar media (DRG International, Inc.; Springfield, NJ, United States) and the *S. mutans*-specific mitis salivarius agar (MSA) (Sigma-Aldrich, United States) supplemented with bacitracin to a final concentration of 0.2 U/mL were used.

### Reagents

RPMI with L-glutamine and HEPES and Concanavalin A-Alexa Fluor 647 fluorescent dye were purchased from Invitrogen (Grand Island, NY, United States); Calcofluor White stain, β-1,3-glucan (laminarin from *Laminaria digitata*), α-mannan (from *Saccharomyces cerevisiae*), lyticase from *Arthrobacter luteus* (β-glucanase) and α-mannosidase (from jack bean) were purchased from Sigma-Aldrich Chemical (St. Louis, MO, United States). *C. albicans* biofilm matrix material (provided by Dr. David Andes) was extracted and purified as previously described (Zarnowski et al., 2016).

### Design and Optimization of High-Content Fluorescence-Based Biofilm Assays

A quantitative biofilm assay based on measurement of fluorescence emission was developed using the GFP-*S. mutans* strain. To standardize fluorescence emission and acquisition, a *S. mutans* cell density-dependent fluorescent assay was performed. *S. mutans* cell suspensions in 10 mM PBS ranging in concentration between 1 × 10^7^−1 × 10^9^ cells/mL were seeded (100 μL) in the wells of transparent flat bottom black sides 96-well plates (Greiner bio-one) and GFP fluorescence was measured at an excitation of 488 nm and emission 530 nm using BioTek cytation 5 (Winooski, VT, United States).

### *In vitro* Analysis of Single and Dual-Species Biofilms

For biofilm formation, 100 μL of 1 × 10^7^ cells/mL *C. albicans* cell suspension in RPMI was added to indicated wells and plates were incubated for 90 min at 37°C. Following incubation, wells were washed with PBS and 100 μL of 1 × 10^4^ cells/mL *S. mutans* cell suspension in RPMI was added into the wells with *C. albicans*. As a control, *S. mutans* was also grown alone in single-species biofilm. Plates were statically incubated at 37°C overnight, and following incubation, the supernatant was discarded, and 100 μL of PBS was added to all wells. Biofilm cells were recovered by sonication (Fisherbrand^TM^ Q500 Sonicator with Probe) (10 s, 0 pulses, 30% ampl), serially diluted in PBS and plated on MSA for CFU (cells/mL) enumeration. Experiments were also performed using the GFP-*S. mutans* strain in single and mixed biofilm assays, and biofilm formation was evaluated by measurement of the fluorescence intensity of the cells recovered after sonication in a water bath (Fisher Scientific Ultrasonic Bath 5.7 L model 15337417) for 20 min at room temperature and processed as described above. Initial experiments were performed to evaluate a potential effect of saliva on microbial adhesion; for these experiments, unstimulated whole human saliva was collected from healthy volunteers using the Salivette system (Sarstedt, Numbrecht, Germany); saliva samples were pooled, clarified by centrifugation (4,500 RPM; 10 min) and filter-sterilized. Prior to biofilm formation, wells were pre-coated with 60 μL of saliva and control wells were coated with PBS. Plates were incubated for 60 min at 37°C then saliva was removed and wells gently washed with 10 mM PBS. Results from these analyses indicated no effect for saliva on adhesion or biofilm formation, therefore, saliva was not used in subsequent experiments.

### Confocal Laser Scanning Microscopy (CLSM) Analysis of Mixed Biofilms

To comparatively visualize the architecture of single and dual-species biofilms, a cell suspension of 1 × 10^7^ cells/mL of *S. mutans* was grown in RPMI alone or added to pre-adhered *C. albicans* in glass-bottom dishes (35 mm petri dish, MatTek Corporation). First, *C. albicans* (1 × 10^7^ cells/mL) was incubated for 90 min at 37°C; and following incubation, dishes were washed with 10 mM PBS, and GFP-*S. mutans* was added, and the dishes were incubated statically at 37°C overnight. The following day, dishes were washed with 10 mM PBS and biofilms were stained with concanavalin-A-Alexa Fluor 647 (0.05 mg/mL) for 45 min at 37°C to stain for extracellular matrix. *C. albicans* hyphae were stained with 0.1% Calcofluor White, which stains cell wall chitin, for 10 min at room temperature. Samples were gently washed with 10 mM PBS and examined by confocal laser scanning microscopy at 40× and 60× magnifications (Nikon Ti2 Spinning Disk). Images were processed using Imaris and Photoshop CS6 software.

### Scanning Electron Microscopy (SEM) Analysis of Mixed Biofilms Formed on Enamel Slabs of Extracted Human Teeth

Extracted sound third molar human teeth were stored at 4°C in a 0.01% (w/v) thymol solution until use. Slabs (4 × 4 × 2 mm) of enamel were generated following root separation using a water-cooled diamond saw (Extec Corp., Enfield, CT, United States) and a cutting machine (IsoMet low-speed saw, Buehler, Lake Buff, IL, United States). Enamel slabs were placed in 24-well plates and pre-coated with unstimulated whole human saliva for 60 min. Following washing with PBS, slabs were immersed in a mixed cell suspension containing 1 × 10^6^ cells/mL for each of *S. mutans*, and *C. albicans* in RPMI and plates were incubated statically for 24 h at 37°C. Following incubation, slabs were gently rinsed in 10 mM PBS and fixed in 2% paraformaldehyde/2.5% glutaraldehyde and following washing steps with PBS, post-fixed with 1% osmium tetroxide; slabs were then rinsed with PBS and dehydrated using a series of washes with ethyl alcohol (30-100%). Samples were dried by critical point drying using an Autosamdri-810 (Tousimius), mounted on aluminum stubs and sputter-coated with 10–20 nm of Platinum/Palladium and imaged with a Quanta 200 scanning electron microscope (FEI Co., Hillsboro, OR, United States).

### Evaluation of Exogenous Supplementation With α-Mannan and β-1,3-Glucan or With *C. albicans* Purified Biofilm Matrix Material on *S. mutans* Adherence and Biofilm Formation

To investigate whether *C. albicans* cell wall polysaccharides augment *S. mutans* biofilm formation, *S. mutans* was grown in media supplemented with purified α-mannan, β-1,3-glucan or purified matrix material from *C. albicans* biofilm extracellular matrix. Mixed biofilms with *C. albicans* with no polysaccharide supplementation were also grown for comparison. For these experiments, individual polysaccharides or matrix material were dissolved in RPMI media to a final concentration of 0.125, 0.25 or 0.5 mg/mL and 50 μL of each solution was added to the indicated wells in a 96-well plate. 100 μL of a 1 × 10^7^ cells/mL *C. albicans* cell suspension in RPMI was incubated in designated wells of the 96-well plate for 90 min at 37°C to serve as positive controls. Subsequently, 50 μL of a 2 × 10^7^ cells/mL GFP-tagged *S. mutans* cell suspension in RPMI was added to each well containing either individual polysaccharides, purified matrix, RPMI only (negative control) or RPMI with pre-adhered *C. albicans* (positive control). Following overnight static incubation at 37°C, the supernatant was removed, cells were covered in 100 μl of PBS and GFP fluorescence was measured as described above. Fluorescence values were evaluated relative to the acquired fluorescence intensity for the *S. mutants* alone control biofilm.

### Evaluation of *S. mutans* Biofilm Formation During Growth in Cell-Free Spent Media From Biofilm Cultures of *C. albicans*

Since *C. albicans* biofilm polysaccharides are secreted into the environment, experiments were performed where spent culture growth media from *C. albicans* 48 h-biofilms were used to grow *S. mutans* biofilm. For these experiments, tissue culture flasks (BioLite 75 cm Flask; Thermo Scientific) were seeded with cells of the *C. albicans* strains to a final cell density of 1 × 10^6^ cells/mL in RPMI, and biofilms were allowed to grow statically at 37°C for 48 h. Spent biofilm media was recovered, filter-sterilized through a 0.22 μm nitrocellulose membrane and diluted (1:1) in fresh RPMI and used to grow *S. mutans* biofilms. To confirm whether the effect of *C. albicans* spent media on *S. mutans* biofilm formation is mediated by the *C. albicans* secreted polysaccharides, recovered spent media was digested with specific enzymes. Additionally, to determine whether proteins in the spent media are also involved, the media was heated at 100°C for 10 min to denature proteins present.

In transparent flat-bottom, black-sides 96-well plates, 50 μL of 2 × 10^7^ cells/mL GFP-*S. mutans* cell suspension in RPMI was added to 50 μL of *C. albicans* cell-free spent media alone or containing pre-diluted enzymes. For enzymatic digestion, a range of enzyme concentrations for α-mannosidase (2, 1, 0.5 U/mL) and β-glucanase (lyticase) (5, 2.5, 1.25 U/mL) were used based on our previous work (Kong et al., 2016). Wells with GFP-*S. mutans* grown alone in RPMI were used as controls. Plates were incubated statically at 37°C for 24 h and samples were processed as described above and GFP fluorescence values were evaluated relative to the acquired fluorescence intensity for the *S. mutants* alone control biofilm.

In a parallel experiment, *S. mutans* suspensions were incubated with the same enzyme dilutions in RPMI to ensure that any observed effect was due to the digestion of spent media contents and not a direct effect of the enzymes on *S. mutans* cells (data not shown).

### CLSM Analysis of *S. mutans* Biofilms Grown in the Presence of Exogenous Supplementation With *C. albicans* Biofilm Matrix Material or in Cell-Free Spent Culture Media

GFP-*S. mutans* biofilms were grown in RPMI supplemented with 0.5 mg/mL of purified matrix from *C. albicans* biofilms in glass-bottom dishes for confocal microscopy analysis. Additionally, *S. mutans* was also grown in spent culture media recovered from *C. albicans* 48 h-biofilms. Following static overnight incubation at 37°C, dishes were washed with 10 mM PBS and biofilms were stained with Concanavalin A-Alexa Fluor 647 (0.05 mg/mL) for 45 min at 37°C. Control wells with *C. albicans-S. mutans* mixed biofilms were additionally stained with calcofluor white (0.1%) for 10 min to visualize *C. albicans*. Following staining, dishes were carefully washed with PBS and CLSM images were taken at 40× and 60× magnifications and images were processed as described above.

### Mouse Model of *C. albicans-S. mutans* Co-infection

All animal experiments were conducted at the AAALAC accredited Animal Facility of the University of Maryland and were approved by the University of Maryland Animal Care and Use Committee (IACUC Protocol #0717010). Two to three-month-old female C57BL/6 mice (Envigo Laboratories, United States) were used in these studies. Animals were divided into three groups: (1) infected only with *S. mutans* (2) infected only with *C. albicans* (3) co-infected with both species. Timeline of infection is illustrated in Figure 6A. Drinking water was supplemented with 0.5 mg/mL ampicillin to control for enteric bacteria due to coprophagy (feces consumption) and mice were immunosuppressed by subcutaneous administration of cortisone acetate (200 mg/kg body weight) every other day starting 1 day before infection to enable *C. albicans* colonization (Solis and Filler, 2012). On the day of *C. albicans* infection, mice were anesthetized using Tribromoethanol (Sigma-Aldrich Co.; 250 mg/kg body weight) solution *via i*ntraperitoneal injections (0.5 mL) and animals were orally infected using calcium alginate swabs (Fisher Scientific) saturated with *C. albicans* cell suspension (2 × 10^7^ cells/mL), which were placed sublingually for 45 min (Figure 6B). The following day, drinking water was replaced with water supplemented with sucrose (final concentration 1% to mimic a cariogenic diet) with no ampicillin and for the next two subsequent days, *S. mutans* cells were freshly added to the drinking water (final cell density of 1 × 10^6^ cells/mL) of groups 1 and 3 on each of the 2 days. To ensure the viability of the bacteria in the drinking water, the water was sampled and cultured for *S. mutans*, which confirmed 100% survival of *S. mutans* for the duration of experiments (data not shown). On day 5, animals in all three groups were euthanized by CO_2_ inhalation followed by cervical dislocation. The oral cavity was clinically evaluated for lesions indicative of oral candidiasis, and teeth and tongues were harvested, weighed, and placed in cold 10 mM PBS. Tongues were homogenized, and teeth were sonicated using a probe sonicator (20 s, 10-s pulses, 60% amplitude) and cell suspensions from both specimens were serially diluted in PBS. All suspensions were cultured in triplicate on yeast chromogenic media and MSA agar for evaluation of *C. albicans* and *S. mutans* recovery, respectively. Plates were incubated for 48 h at 37°C, and viable counts were enumerated and expressed as log_10_ CFUs/gram tissue.

#### Tissue Histopathology Analyses of Tongue Tissue

In order to visually assess the fungal presence and tissue invasion, representative tongues from all groups were fixed in paraformaldehyde, embedded in paraffin and sectioned; tissue sections were deparaffinized with xylene and stained with Periodic Acid Schiff (PAS) to highlight *C. albicans* hyphae. The whole periphery of each infected tongue section was examined by light microscopy and evaluated based on the presence and extent of adhering yeast cells and penetration of the epithelium by invasive hyphae.

#### Scanning Electron Microscopy (SEM) of Infected Tongue Tissue and Teeth Surface

Representative tongues and teeth from mice from all groups were subjected to SEM analysis to visualize biofilms formed on oral surfaces. Samples were fixed and processed as described above.

### Statistical Analysis

All statistical analyses were carried out using Prism software (GraphPad, United States). Different sample groups were analyzed by unpaired *t*-test or one-way ANOVA. Differences were considered statistically significant if *p*-values < 0.05. All *in vitro* experiments were performed in at least triplicates, in no less than three separate occasions. Animal experiments were performed on four separate occasions, with 3–5 animals per group.

## Results

### Comparative Analysis of Single and Dual-Species Biofilms *in vitro*

**FIGURE 1:**
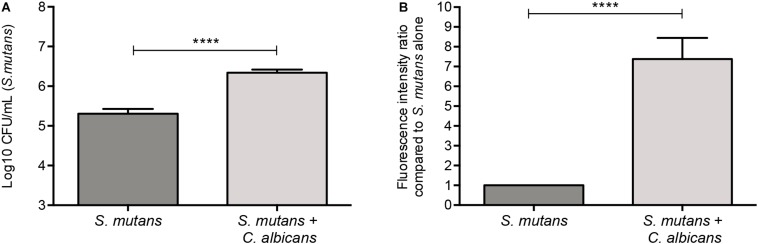
Significantly higher *S. mutans* recovery from mixed biofilms with *C. albicans*. **(A)** Based on CFU (cells/ml) counts, significantly higher level of *S. mutans* is recovered from mixed biofilms compared to single biofilm. **(B)** Similar results were seen when biofilms were evaluated based on GFP fluorescence intensity (arbitrary units) of *S. mutans*-GFP cells. Unpaired *t*-test; **p = 0.0053, ****p ≤ 0.0001.

**FIGURE 2:**
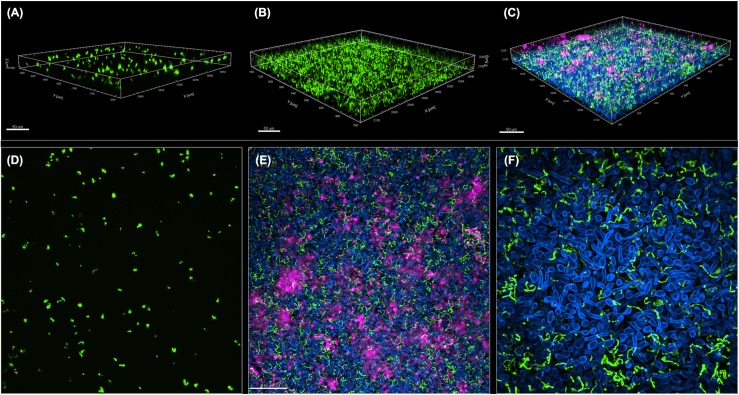
Comparative CLSM analysis of *S. mutans* single and mixed 24-hr biofilms with *C. albicans*. **(A,D)** GFP-*S. mutans* single-species biofilms demonstrating a sparse biofilm with small cell aggregates. **(B,C,E,F)** In contrast, growth of GFP-*S. mutans* with *C. albicans* resulted in the formation of a dense and complex biofilm. **(A–C)** Representative 3D images showing biofilm distribution for **(A)** GFP-*S. mutans* alone, **(B)** GFP-*S. mutans* channel in a mixed biofilm with *C. albicans* and **(C)** GFP-*S. mutans* and *C. albicans* in a mixed biofilm. **(E,F)** Representative Z-stacks showing adherence and clumping of bacterial cells around the hyphae. GFP-*S. mutans* (green); *C. albicans* cell wall chitin stained with Calcofluor White (blue); Extracellular matrix of biofilm stained with Concanavalin-A-Alexa Fluor 647 (magenta). Images were acquired using Spinning disk confocal Nikon Ti2 inverted microscope and processed with Imaris software.

**FIGURE 3:**
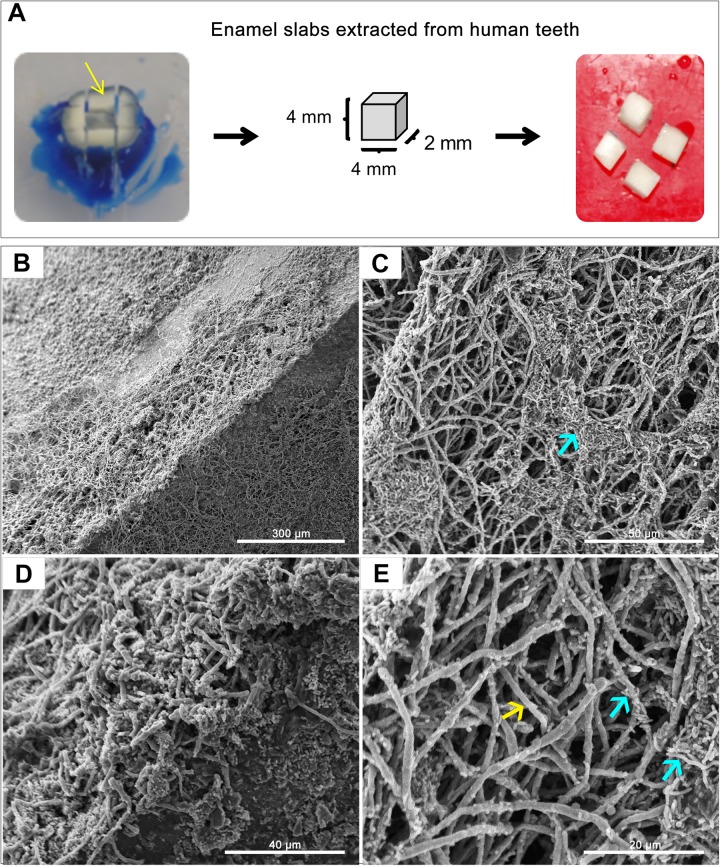
Representative Scanning Electron Microscopy (SEM) micrographs of *in vitro*-grown dual-species biofilms on slabs of human teeth. **(A)** Dentin and enamel slabs were obtained from intact 3rd molar teeth using a water-cooled diamond saw and a cutting machine. Mixed biofilms were grown on slabs for 24 h at 37°C. **(B–E)** SEM micrographs demonstrating a robust and thick mixed biofilm formed on the slabs consisting of intertwined hyphal matrix with aggregates of bacteria (blue arrows) formed around the hyphae (yellow arrow).

A GFP-producing *S. mutans* strain was used to develop a novel assay to assess biofilm formation based on fluorescence emission measurement. A standard curve consistently demonstrated an *S. mutans* cell-density dependent increase in fluorescence signal indicating the sensitivity of the assay for use as a high-throughput quantitative assay for evaluation of *S. mutans* biofilm formation (Supplementary Figure S1). Additionally, *S. mutans* recovery from single and mixed-species biofilms was also comparatively assessed based on colony-forming units (CFU) counts; results demonstrated significantly enhanced *S. mutans* recovery from mixed biofilms with *C. albicans* compared to *S. mutans* single-species biofilms. CFU results were consistent with those from the fluorescent-based biofilm assay (Figure 1). Initial experiments were performed to evaluate a potential effect of saliva on microbial adhesion, and results from these analyses indicated no effect for saliva on adhesion or biofilm formation, therefore, saliva was not used in subsequent experiments (data not shown). Similarly, Confocal Laser Scaning Microscopy (CLSM) analysis of formed biofilms revealed that, compared to *S. mutans* single biofilm, growth with *C. albicans* resulted in a significant increase in biofilm biomass and complexity concurrent with a significantly increased level of *S. mutans* retention (Figures 2B,D,F). In addition, *S. mutans* cells could be seen adhering to *C. albicans* yeast cells and hyphae, specifically forming dense cell aggregates around the hyphae (Figure 2F). In stark contrast, sparse distribution of *S. mutans* cells was seen in the single species biofilm, which comprised of individual cells and small clumps (Figures 2A,C,E). To further explore the clinical relevance, mixed species biofilms were grown on saliva-coated enamel slabs from extracted human teeth and analyzed by Scanning Electron Microscopy (SEM) (Figure 3A). Consistent with findings from microbial recovery and CLSM imaging, SEM micrographs revealed avid adherence of *S. mutans* to *C. albicans* forming a mature and thick biofilm consisting of *C. albicans* hyphae, *S. mutans* aggregates and polysaccharide matrix (Figures 3B–E).

### Evaluation of Exogenous Supplementation With α-Mannan, β-1,3-Glucan, and Purified *C. albicans* Matrix Material on *S. mutans* Biofilm Formation

**FIGURE 4:**
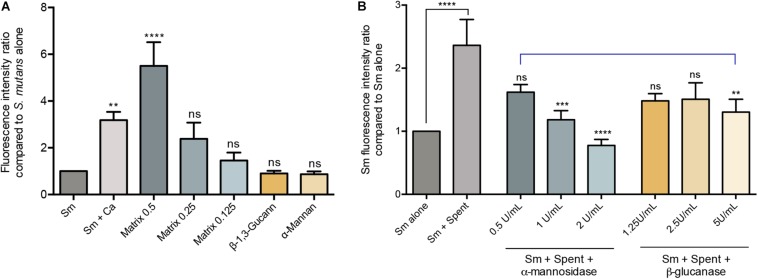
Evaluation of the various *C. albicans* effectors mediating mixed biofilm formation. **(A)** Based on measurement of fluorescence emitted from GFP-*S. mutans*, significantly higher fluorescence intensity was detected in mixed biofilms (Sm+Ca) compared to that in *S. mutans* single biofilm (Sm); similarly, matrix supplementation resulted in a matrix dose-dependent increase in fluorescence, but not supplementation with β-Glucan or α-Mannan (0.5 mg/mL, both). Matrix concentrations tested included 0.5, 1.25, and 0.125 mg/mL. **(B)** To evaluate the role of *C. albicans* secreted matrix polysaccharides in mediating *S. mutans* retention, *S. mutans* single biofilms were grown in cell-free spent media from the *C. albicans* strains. Based on measurement of emitted fluorescence from 24 h *S. mutants* biofilms, significantly higher *S. mutans* signal was detected from biofilms grown in spent media (Sm+Spent) compared to that from the *S. mutans* alone. The observed increase in emitted fluorescence from *S. mutans* biofilms grown in *C. albicans* spent media was significantly diminished upon enzymatic digestion of the secreted polysaccharides in the media by α-mannosidase (0.5, 1, and 2 U/mL) and β-glucanase (1,25, 2.5, and 5 U/mL) enzymes with α-mannosidase treatment being more impactful. Statistical comparison between *Sm* alone and *Sm* + spent is indicated with a black bar. Comparative analysis between *Sm* biofilm in digested-spent media versus *Sm* + spent is indicated with a blue bar. ns: not significant; **p ≤ 0.01; ***p ≤ 0.005; ****p ≤ 0.001.

The polysaccahrides α-mannan, and β-1,3-glucan as well as purified matrix material from *C. albicans* biofilms were used in biofilm assays to determine the effect of these polysaccharides on *S. mutans* biofilm formation. Based on quantitative fluorescence measurement, results demonstrated that *S. mutans* growth in the presence of 0.5 mg/mL of α-mannan or β-1,3-glucan was comparable to that from *S. mutans* biofilms with no polysaccharide supplementation. In contrast, however, *S. mutans* recovery from biofilms supplemented with the *C. albicans* purified biofilm matrix was significantly increased, and was comparable or higher than *S. mutans* recovery from dual-species biofilms at 0.25 and 0.5 mg/mL, respectively (Figure 4A).

### Evaluation of *S. mutans* Biofilm Formation During Growth in Cell-Free Spent Culture Media From *C. albicans*

Since polysaccharides are components of the fungal cell wall and are secreted into the environment, experiments were designed where biofilm culture media from *C. albicans* was collected and used to grow *S. mutans* biofilms. Results from these experiments demonstrated significantly increase of *S. mutans* recovery in biofilms grown in the spent media compared to *S. mutans* grown alone (Figure 4B). The critical role for the secreted polysaccharides in the spent culture media was further confirmed using enzymatic digestion, where α-mannosidase and lyticase (glucanase) enzymes that degrade mannan and glucans, respectively, were incorporated in the *S. mutans* biofilm formation assays. The results from the enzymatic digestion demonstrated a significant reduction in *S. mutans* recovery from biofilms grown in spent media where α-mannan components were digested, compared to biofilms grown in undigested spent media (Figure 4B), but a modest reduction was observed when β-1,3-glucan components were digested. To rule out a contribution from secreted proteins, spent media was heated to denature any proteins; results from these experiments indicated a minimal and insignificant role for *C. albicans* secreted proteins in the *S. mutans-C. albicans* interaction (data not shown).

### CSLM Analysis of *S. mutans* Biofilms Grown in *C. albicans* Cell-Free Spent Media or in the Presence of *C. albicans* Purified Biofilm Matrix

**FIGURE 5:**
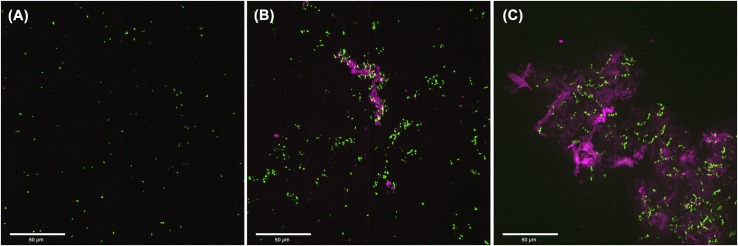
Confocal Laser Scanning Microscopy (CLSM) analysis of *S. mutans* biofilms grown in *C. albicans* cell free spent media or media supplemented with purified extracellular matrix. **(A)** 24-hr single biofilms of GFP-*S. mutans* appeared sparse with minimal cells or cell aggregates seen. **(B)** Significantly more *S. mutans* cells was seen when *S. mutans* was grown in *C. albicans* spent media with considerable amount of bacterial clumping around secreted polysaccharide aggregates. **(C)** *S. mutans* biofilm grown in the presence of purified extracellular matrix from *C. albicans* biofilms showing large aggregates of polysaccharides with embedded aggregates of bacteria. Pink: polysaccharides stained with Con-A; Green: GFP-*S. mutans*.

To visualize the structure of *S. mutans* biofilms formed under conditions associated with *C. albicans* effectors, CSLM was used. Images revealed a complex biofilm consisting of aggregating bacterial cells embedded in extracellular matrix, when *S. mutans* was grown in *C. albicans* spent media (Figure 5B) or with purified *C. albicans* matrix material (Figure 5C). In contrast, few aggregates of *S. mutans* were visible in biofilms grown in non-supplemented media (Figure 5A).

### *C. albicans*-Mediated Enhanced *S. mutans* Colonization *in vivo*

**FIGURE 6:**
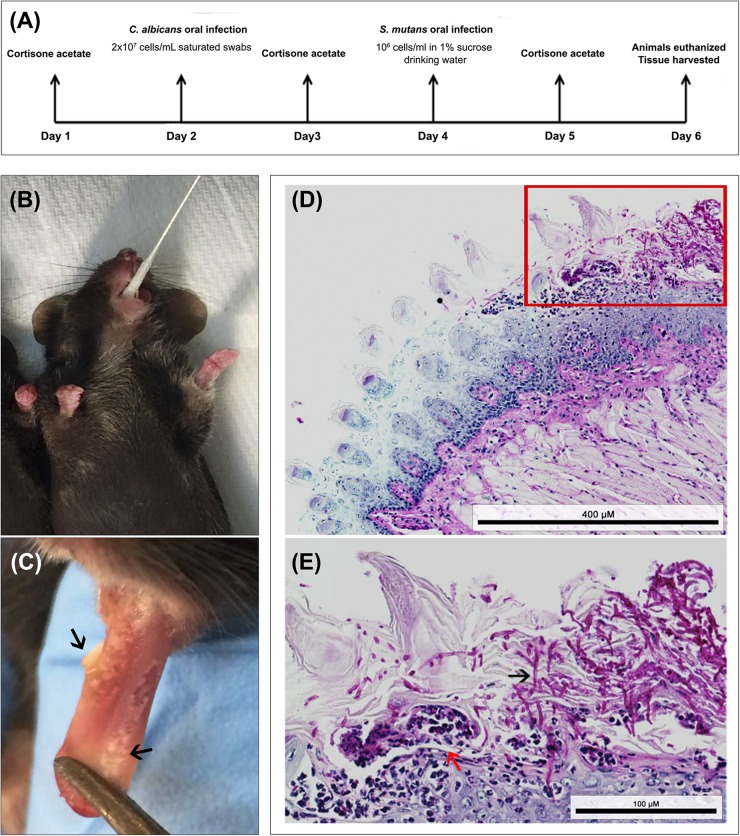
Animal model of oral co-infection. **(A)** Infection protocol and timeline; animals were euthanized and infection assessed 4 days post-infcetion with *C. albicans*. **(B)** Representative image demonstrating mouse sublingual oral infection using calcium alginate swabs saturated with *C. albicans* cell suspension. **(C)** Representative image of a tongue from an infected animal with multiple white lesions typical of oral candidiasis (black arrows). **(D,E)** Representative images from histopathology analysis of PAS stained tongue tissue sections from co-infected mice demonstrating extensive presence of *C. albicans* around the periphery of the tongue along with hyphal invasion (black arrows) into the sub-epithelial tissue; influx of neutrophils (red arrow) indication local immune response to the infection. Bacterial cells are not visible with PAS stain. Scale bar 400 μm; Inset scale bar 100 μm.

**FIGURE 7:**
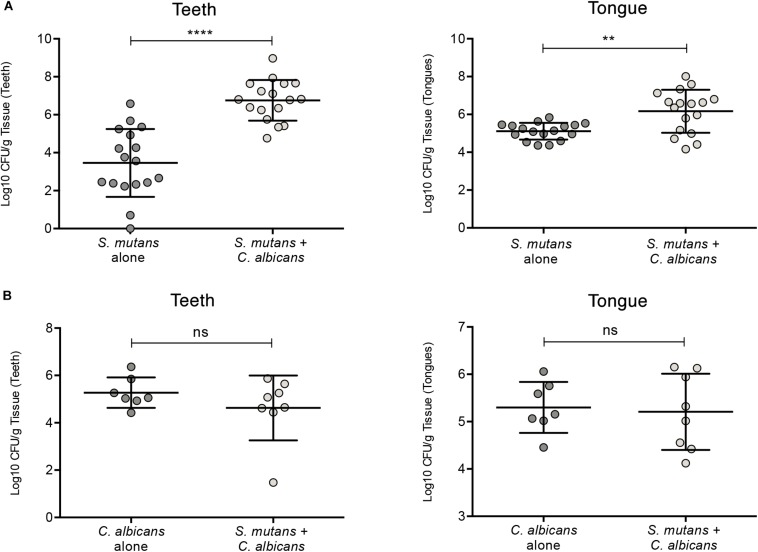
Evaluation of *S. mutans* and *C. albicans* recovery from teeth and tongues of mice infected with *S. mutans* and *C. albicans* individually or in combination. **(A)** Significantly higher level of *S. mutans* recovery from teeth and tongues of co-infected animals compared to animals infected only with *S. mutans*. **(B)** No difference in the recovery of *C. albicans* from teeth and tongues from co-infected animals compared to animals infected only with *C. albicans*. ns: not significant; *p ≤ 0.05.

**FIGURE 8:**
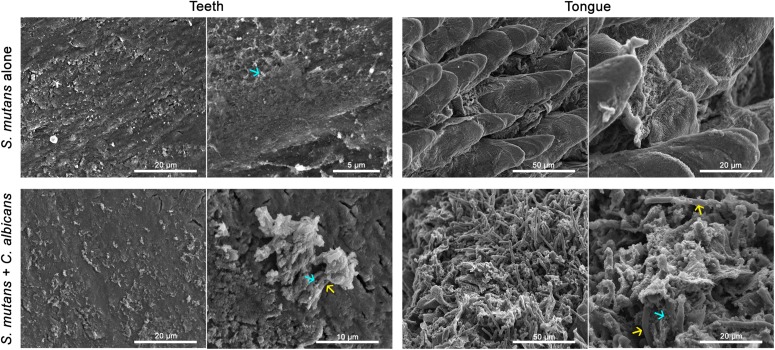
Comparative SEM analysis of teeth and tongues from *S. mutans*-infected and co-infected animals. Enhanced *S. mutans* colonization of oral samples from co-infected animals compared to *S. mutans*-infected animals. Teeth of *S. mutans*-only infected mouse showing scattered clumps of *S. mutans* compared to teeth of co-infected mouse demonstrating a thicker biofilm of *C. albicans* intermixed with *S. mutans* embedded in extracellular matrix material. Tongue of *S. mutans*-only infected mouse with minimal *S. mutans* adherence and biofilm formation; in contrast to tongue of co-infected mouse with oral candidiasis with massive hyphal invasion of oral tissue. A thick biofilm is seen consisting of *C. albicans* and *S. mutans* adhering avidly to the surface of *C. albicans* and within clumps of extracellular matrix. blue arrows: *S. mutans*, yellow arrows: *C. albicans*.

**FIGURE 9:**
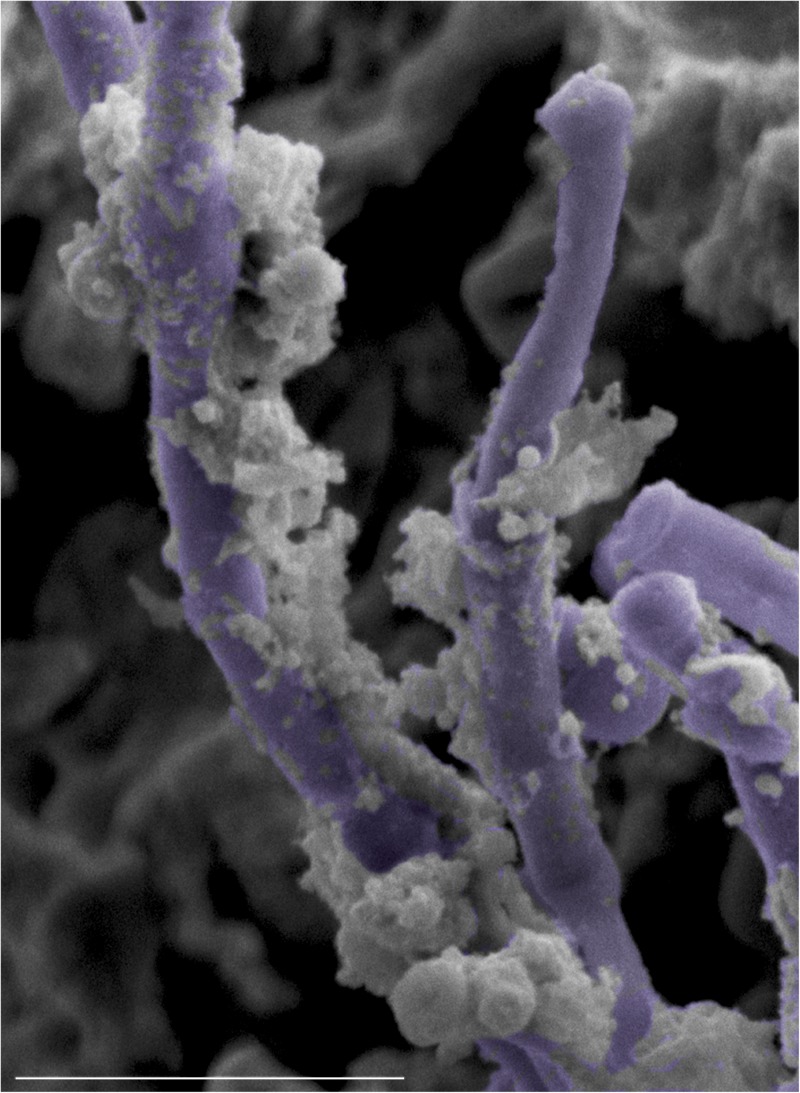
High magnification SEM micrograph of the surface of a tongue from a co-infected mouse with oral candidiasis demonstrating *S. mutans* adhering avidly to the surface of *C. albicans* hyphae (pseudo-colored in purple). Bar = 10 μm.

To test whether *C. albicans* can enhance *S. mutans* colonization in a host, we optimized an oral co-infection mouse model, based on our established mouse model of oral candidiasis (Kong et al., 2015). Upon clinical evaluations of oral tissue, evident and comparable lesions indicative of oral candidiasis were observed in all mice infected with *C. albicans* (alone or with *S. mutans*) (Figure 6C). Based on microbiological analysis of harvested teeth and tongues from all groups, significantly higher level of *S. mutans* was recovered from co-infected mice compared to *S. mutans*-only infected mice (Figure 7A). However, no significant differences in *C. albicans* recovery were seen from tongues and teeth of co-infected compared to *C. albicans*-only infected mice (Figure 7B). Consistent with clinical picture, histopathological evaluation of tongue tissue sections from all animals with oral candidiasis revealed penetration of the invasive hyphae into the epithelial tissue (Figures 6D,E). Similarly, SEM imaging of harvested tongues and teeth demonstrated the presence of a complex mixed-species biofilm with hyphae seen penetrating the oral tissue along with bacteria colonizing the tissue and co-adhering to the hyphae (Figures 8, 9). In contrast, minimal presence of bacteria was seen colonizing tongues and teeth of animals infected only with *S. mutans* (Figure 8).

## Discussion

The secreted extracellular matrix of both *S. mutans* and *C. albicans* is essential for the retention and interaction of these species within a biofilm (Koo et al., 2018). In addition to fermentation of sucrose and other carbohydrates to generate lactate, *S. mutans* also uses sucrose as a substrate for exopolysaccharides synthesis (Xiao et al., 2012). The *S. mutans* secreted polysaccharides β-1,3-glucans and β-1,6-glucans synthesized by glucosyltransferases (Gtfs), are the main components of the *S. mutans* biofilm extracellular matrix (Bowen and Koo, 2011; Gregoire et al., 2011). Interestingly, the *S. mutans*-derived GtfB enzyme was shown to bind to *C. albicans* cell wall α-mannan contributing to the formation of dual-species biofilm (Hwang et al., 2015; Hwang et al., 2017; Kim et al., 2018). Furthermore, in addition to mediating co-adherence, *S. mutans* GtfB activity was also hypothesized to provide carbohydrates which serve as a source of nutrient for *C. albicans* (Falsetta et al., 2014). The importance of streptococci glucosyltransferases was similarly indicated by findings from a study identifying a pivotal role for the *Streptococcus gordonii* GtfG enzyme in promoting binding of the bacteria to *C. albicans*, and in enhancing dual biofilm formation (Ricker et al., 2014).

Similar to *S. mutans*, *C. albicans* secreted polysaccharides are important components of the fungal biofilm matrix, with α-mannan being the most abundant component followed by β-1,6-glucan and β-1,3-glucan (Zarnowski et al., 2014; Pierce et al., 2017). However, the presence of one secreted *C. albicans* exopolysaccharide was shown to be required for the assembly of the other exopolysaccharides, and the lack of any of the exopolysaccharides reduced the abundance of all three (Mitchell et al., 2015). This outcome was evidenced by findings demonstrating that growth of *C. albicans* strains with modulated secreted polysaccharides production together with wild-type strains resulted in compensation of the extracellular exopolysaccharides (Mitchell et al., 2015). Most importantly, the secreted α-mannan, β-1,6-glucan, and β-1,3-glucan which make up the *C. albicans* biofilm matrix, are structurally different from *C. albicans* cell wall polysaccharides (Mitchell et al., 2015).

Our *in vitro* findings clearly demonstrated higher mixed biofilm biomass in *S. mutans-C. albicans* biofilms compared to *S. mutans* single species biofilm (Lobo et al., 2019). This was expected given the complex hyphal matrix, which is the hallmark of *C. albicans* biofilm, as demonstrated by SEM micrographs of mixed biofilms grown on enamel slabs of extracted human teeth (Figures 3B–E). Although saliva is considered to play an essential role in mediating microbial interactions in the oral cavity, in our experiments, precoating surfaces with human saliva had minimal impact on *S. mutans* adherence under all conditions tested (data not shown). These observations are consistent with those from a previous study similarly demonstrating no difference in the adhesion of *S. mutans* or *C. albicans* to surfaces precoated or not coated with saliva (Izumida et al., 2014).

Analysis of mixed biofilms using confocal fluorescent imaging indicated a central role for the *C. albicans* secreted polysaccharides in mediating adherence and dual-species biofilm formation (Figure 2). Therefore, given the high abundance of α-mannan in *C. albicans* biofilm matrix and the significant role of β-1,3-glucan in the mannan–glucan complex, to isolate the contribution of these polysaccharides we performed *S. mutans* biofilm assays with exogenous supplementation of α-mannan and β-1,3-glucan. More importantly, similar experiments were performed incorporating purified whole matrix material derived from *C. albicans* biofilm (Zarnowski et al., 2016). The combined findings demonstrated that the presence of α-mannan and β-1,3-glucan individually had little impact on *S. mutans* biofilm formation. In contrast, supplementation with purified whole matrix material from *C. albicans* biofilms resulted in the formation of a dense *S. mutans* biofilm comparable to the mixed *S. mutans-C. albicans* biofilms (Figure 4A). Combined, these findings strongly indicate that a complex mixture of all *C. albicans* polysaccharide components is needed to impact *S. mutans* biofilm formation, and that secreted polysaccharides have a higher impact on *S. mutans* biofilm formation than the presence of *C. albicans.*

In addition to exogenous polysaccharides, *S. mutans* biofilms were grown in cell-free spent *C. albicans* biofilm culture media containing secreted polysaccharides among other polymers, to isolate the contribution of secreted polysaccharides specifically. Based on microscopic and microbial recovery evaluations, results demonstrated that when grown in *C. albicans* spent media, *S. mutans* formed significantly enhanced biofilms (Figure 4B). However, *S. mutans* recovery was not increased when biofilms were formed in spent media in the presence of mannan and glucan-digesting enzymes, with notably less prominent effect for glucan digestion. These findings are consistent with those from a study by Hwang et al. (2017) exploring the contribution of *S. mutans* GtfB in mediating mixed biofilm formation. Using *C. albicans* strains defective in *O*-mannan, the findings from the study demonstrated that cell wall mannans mediate *S. mutans* GtfB binding to *C. albicans*, and modulate this inter-species interaction in a rodent biofilm model. In our study, however, by using purified polysaccharide material from *C. albicans* biofilm matrix, as well as spent media, we demonstrated that *C. albicans* secreted effectors are sufficient to enhance *S. mutans* biofilm formation irrespective of the presence of the fungal cells (Figures 4A,B, 5). Significantly, these findings may have clinical relevance as a recent study by Montelongo-Jauregui et al. (2019) demonstrated that *C. albicans* mutant strains deficient in matrix production are more susceptible to antimicrobials when grown in biofilm with *S. gordonii.* The use of *C. albicans* mutant strains lacking key genes involved in the synthesis and secretion of mannan would confirm the role of the secreted mannan and mannan-complexes on *S. mutans* growth in mixed biofilms. However, as described by others and confirmed by us, the key available mutants are aberrant in adhesion, hyphal and biofilm formation and virulence due to pronounced structural changes in their cell walls (Southard et al., 1999; Munro et al., 2005; Mitchell et al., 2015; Lee et al., 2016). Therefore, given these limitations, it was not possible to use these mutants to confidently determine the impact of the absence of the relevant genes on *S. mutans* adherence and retention in a mixed biofilm.

To demonstrate the phenomenon of *C. albicans*-mediated enhanced *S. mutans* colonization in an environment that anatomically and immunologically mimics humans, a mouse model of oral co-infection was optimized based on our established mouse model of oral candidiasis (Kong et al., 2015). In our model, the animals were exposed to *S. mutans* through drinking water supplemented with 1% sucrose post-infection with *C. albicans*, as our hypothesis is that colonization with *C. albicans* and onset of candidiasis augment and support *S. mutans* retention and colonization. Microbiological analysis of teeth and tongues harvested from euthanized animals demonstrated significantly enhanced *S. mutans* recovery from teeth of co-infected mice, compared to those from mice exposed only to *S. mutans* (Figure 7A). In contrast, however, no significant differences in the extent of clinical lesions, or *C. albicans* recovery was observed between co-infected mice and *C. albicans* only infected mice, indicating that *S. mutans* does not modulate *C. albicans* colonization, or exacerbate *C. albicans* infection (Figure 7B).

As early colonizers of the human oral cavity, streptococci are considered important in establishing *C. albicans* colonization (Xu and Dongari-Bagtzoglou, 2015). However, in our experiments, *C. albicans* recovery from tongues and teeth of infected mice was comparable in the absence and presence of *S. mutans* (Figure 7B). It is important to note that in our model, *S. mutans* was introduced after *C. albicans* colonization and infection were established and therefore, under our experimental conditions, the presence of *S. mutans* in the oral cavity would not be a factor in modulating *C. albicans* recovery. Interestingly though, recent studies by Xu et al. (2016) exploring the impact of *S. oralis* on *C. albicans* pathogenesis in a mouse model, demonstrated that co-infection with *S. oralis* augmented *C. albicans* pathogenicity by amplifying the mucosal inflammatory response, as well as augmenting *C. albicans* invasion through epithelial junctions (Xu et al., 2014; Xu et al., 2016). In our hands, based on clinical and histopathological evaluations, we did not observe an effect for *S. mutans* in enhancing *C. albicans* pathogenesis.

## Conclusion

In conclusion, this study provides mechanistic insights furthering our understanding of the synergistic interactions between *S. mutans* and *C. albicans* and their potential impact on oral disease development, in particular, dental caries. In light of these findings, future efforts should focus on developing therapeutic strategies with targeted actions geared toward prevention *via* manipulation of adhesion receptors to impede the development of disease. However, in-depth studies are warranted to determine mechanistically precise details of adhesion and signaling under conditions of co-existence in the host. Until then, based on the mounting evidence from clinical studies, the presence of *C. albicans* in the oral environment should be taken into account in evaluating risks for dental caries.

## Data Availability Statement

The datasets generated for this study are available on request to the corresponding author.

## Ethics Statement

The animal study was reviewed and approved by all animal experiments were conducted at the AAALAC accredited Animal Facility of the University of Maryland and were approved by the University of Maryland Animal Care and Use Committee (IACUC Protocol #0717010).

## Author Contributions

ZK, TV, TP, AS, and MM conceived, designed and performed the experiments. ZK, TV, and MJ-R analyzed the data. ZK, TV, DM-J, and MJ-R drafted the manuscript. All authors approved the final version of the manuscript.

## Conflict of Interest

The authors declare that the research was conducted in the absence of any commercial or financial relationships that could be construed as a potential conflict of interest.

## References

[B1] Al-Fattani M. Douglas L. (2006). Biofilm matrix of *Candida albicans* and *Candida tropicalis*: chemical composition and role in drug resistance. *J. Med. Microbiol.* 55 999–1008. 10.1099/jmm.0.46569-0 16849719

[B2] Barbieri D. Vicente V. Fraiz F. Lavoranti O. Svidzinski T. Pinheiro R. (2007). Analysis of the *in vitro* adherence of *Streprococcus mutans* and *Candida albicans*. *Braz. J. Microbiol.* 38 624–663.

[B3] Bertolini M. Ranjan A. Thompson A. Diaz P. I. Sobue T. Maas K. (2019). *Candida albicans* induces mucosal bacterial dysbiosis that promotes invasive infection. *PLoS Pathog.* 15:e1007717. 10.1371/journal.ppat.1007717 31009520 PMC6497318

[B4] Bowen W. Koo H. (2011). Biology of S*treptococcus mutans*-derived glucosyltransferases: role in extracellular matrix formation of cariogenic biofilms. *Caries Res.* 45 69–86. 10.1159/000324598 21346355 PMC3068567

[B5] de Carvalho F. G. Silva D. S. Hebling J. Spolidorio L. C. Spolidorio D. M. P. (2006). Presence of mutans streptococci and *Candida* spp. in dental plaque/dentine of carious teeth and early childhood caries. *Arch. Oral. Biol.* 51 1024–1028. 10.1016/j.archoralbio.2006.06.001 16890907

[B6] Diaz P. I. Xie Z. Sobue T. Thompson A. Biyikoglu B. Ricker A. (2012). Synergistic interaction between *Candida albicans* and commensal oral streptococci in a novel in vitro mucosal model. *Infect. Immun.* 80 620–632. 10.1128/IAI.05896-11 22104105 PMC3264323

[B7] Duran-Pinedo A. E. Frias-Lopez J. (2015). Beyond microbial community composition: functional activities of the oral microbiome in health and disease. *Microbes Infect.* 17 505–516. 10.1016/j.micinf.2015.03.014 25862077 PMC4495649

[B8] Calderone R. E. (ed.) (2012). *Candida and Candidiasis.* Washington, DC: ASM Press.

[B9] Eidt G. Andrade C. G. Negrini T. C. Arthur R. A. (2019). Role of *Candida albicans* on enamel demineralization and on acidogenic potential of *Streptococcus mutans in vitro* biofilms. *J. Appl. Oral. Sci.* 9:e20180593. 10.1590/1678-7757-2018-0593 31508792 PMC9648952

[B10] Ellepola K. Liu Y. Cao T. Koo H. Seneviratne C. (2017). Bacterial GtfB augments *Candida albicans* accumulation in cross-kingdom biofilms. *J. Dent. Res.* 96 1129–1135. 10.1177/0022034517714414 28605597 PMC5582686

[B11] Falsetta M. L. Klein I. Colone P. M. Scott-Anne K. Gregoire S. Pai C.-H. (2014). Symbiotic relationship between *Streptococcus mutans* and *Candida albicans* synergizes the virulence of plaque-biofilms *in vivo*. *Infect. Immun.* 82 1968–1981. 10.1128/IAI.00087-14 24566629 PMC3993459

[B12] Fidel P. L. J. (2011). Candida-host interactions in HIV disease implications for oropharyngeal candidiasis. *Adv. Dent. Res.* 23 45–49. 10.1177/0022034511399284 21441480 PMC3144040

[B13] Fragkou S. Balasouli C. Tsuzukibashi O. Argyropoulou A. Menexes G. Kotsanos N. (2016). S*treptococcus mutans*, *Streptococcus sobrinus* and *Candida albicans* in oral samples from caries-free and caries-active children. *Eur. Arch. Paediatr Dent.* 17 367–375. 10.1007/s40368-016-0239-7 27357362

[B14] Gillum A. M. Tsay E. Y. Kirsch D. R. (1984). Isolation of the *Candida albicans* gene for orotidine‘5’-phosphate decarboxylase by complementation of *S. cerevisiae* ura3 and *E. coli* pyrF mutations. *Molec. Gen. Genet.* 198 179–182. 10.1007/bf00328721 6394964

[B15] Gregoire S. Xiao J. Silva B. B. Gonzalez I. Agidi P. S. Klein M. I. (2011). Role of glucosyltransferase B in interactions of *Candida albicans* with *Streptococcus mutans* and with an experimental pellicle on hydroxyapatite surfaces. *Appl. Environ. Microbiol.* 77 6357–6367. 10.1128/AEM.05203-11 21803906 PMC3187131

[B16] Hubertine M. W. Kos K. Jabra-Rizk M. A. Korm B. P. (2016). *Candida albicans* in oral biofilms could prevent caries. *Pathog. Dis.* 74:ftw039. 10.1093/femspd/ftw039 27129365

[B17] Hwang G. Liu Y. Kim D. Li Y. Krysan D. J. Koo H. (2017). Candida albicans mannans mediate *Streptococcus mutans* exoenzyme GtfB binding to modulate cross-kingdom biofilm development *in vivo*. *PLoS Pathog.* 13:e1006407. 10.1371/journal.ppat.1006407 28617874 PMC5472321

[B18] Hwang G. Marsh G. Gao L. Waugh R. Koo H. (2015). Binding force dynamics of *Streptococcus mutans*–glucosyltransferase B to *Candida albicans*. *J. Dent. Res.* 94 1310–1317. 10.1177/0022034515592859 26138722 PMC4547317

[B19] Isalm B. Khan S. Khan A. U. (2007). Dental caries: from infection to prevention. *Med. Sci. Monit.* 13 RA196–RA203. 17968308

[B20] Izumida F. E. Moffa E. B. Vergani C. E. Machado A. L. Jorge J. H. Giampaolo E. T. (2014). *In vitro* evaluation of adherence of *Candida albicans*, *Candida glabrata*, and *Streptococcus mutans* to an acrylic resin modified by experimental coatings. *Biofouling* 30 525–533. 10.1080/08927014.2014.894028 24684564

[B21] Jabra-Rizk M. A. Falkler W. A. Jr. Meiller T. F. (2004). Fungal biofilms and drug resistance. *Emerg. Infect. Dis.* 10 14–19. 15078591 10.3201/eid1001.030119 PMC3031105

[B22] Jabra-Rizk M. A. Kong E. Tsui C. Nguyen M. Clancy C. Fidel P. (2016). *Candida albicans* pathogenesis: fitting within the host-microbe damage response framework. *Infect. Immun.* 84 2724–2739. 10.1128/IAI.00469-16 27430274 PMC5038058

[B23] Jarosz L. M. Deng D. van der Mei H. Crielaard W. Krom B. (2009). *Streptococcus mutans* competence-stimulating peptide inhibits *Candida albicans* hypha formation. *Eukaryot. Cell* 8 1658–1664. 10.1128/EC.00070-09 19717744 PMC2772401

[B24] Jenkinson H. F. Barbour M. E. Jagger D. C. Miles M. Bamford C. M. Nobbs A. H. (2008). *Candida albicans* – bacteria interactions in biofilms and disease. *Univ. Bristol Dent. Sch.* 1–6.

[B25] Jenkinson H. F. Lala H. C. Shepherd M. G. (1990). Coaggregation of *Streptococcus sanguis* and other streptococci with *Candida albicans*. *Infect. Immun.* 58 1429–1436. 10.1128/iai.58.5.1429-1436.1990 2182544 PMC258643

[B26] Jenkinson H. F. Lamont R. J. (2005). Oral microbial communities in sickness and in health. *Trends Microbiol.* 13 589–595. 10.1016/j.tim.2005.09.006 16214341

[B27] Kidd E. A. M. Fejerskow O. (2004). What constitutes dental caries? Histopathology of carious enamel and dentin related to the action of cariogenic biofilms. *J. Den. Res.* 83 C35–C38. 15286119 10.1177/154405910408301s07

[B28] Kim D. Liu Y. Benhamou I. Sanchez H. Simón-Soro Á Li Y. (2018). Bacterial-derived exopolysaccharides enhance antifungal drug tolerance in a cross-kingdom oral biofilm. *ISME J.* 12 1427–1442. 10.1038/s41396-018-0113-1 29670217 PMC5955968

[B29] Klein M. I Hwang G. Santos P. H. Campanella O. H. Koo H. (2015). *Streptococcus mutans*-derived extracellular matrix in cariogenic oral biofilms. *Front. Cell. Infect. Microbiol.* 5:10 10.3389/fcimb.2015.00010 PMC4327733 25763359

[B30] Klinke T. Guggenheim B. Klimm W. Thurnheer T. (2011). Dental caries in rats associated with *Candida albicans*. *Caries Res.* 45 100–106. 10.1159/000324809 21412001

[B31] Kolenbrander P. E. Anserson R. X. Blehert D. S. Egland P. G. Foster J. S. Palmer R. J. Jr. (2002). Communication among oral bacteria. *Microbiol. Mol. Biol. Rev.* 66 486–505. 12209001 10.1128/MMBR.66.3.486-505.2002 PMC120797

[B32] Kong E. Kucharíková S. Van Dijck P. Peters B. Shirtliff M. Jabra-Rizk M. (2015). Clinical implications of oral candidiasis: host tissue damage and disseminated bacterial disease. *Infect. Immun.* 83 604–613. 10.1128/IAI.02843-14 25422264 PMC4294238

[B33] Kong E. Tsui C. Kucharíková S. Andes D. Van Dijck P. Jabra-Rizk M. A. (2016). Commensal protection of *Staphylococcus aureus* against antimicrobials by *Candida albicans* biofilm matrix. *mBio* 7:e01365-16. 10.1128/mBio.01365-16 27729510 PMC5061872

[B34] Koo H. Andes D. R. Krysan D. J. (2018). *Candida*–streptococcal interactions in biofilm-associated oral diseases. *PLoS Pathog.* 14:e1007342. 10.1371/journal.ppat.1007342 30543717 PMC6292568

[B35] Koo H. Bowen W. H. (2014). *Candida albicans* and *Streptoc*occus mutans: a potential synergistic alliance to cause virulent tooth decay in children. *Future Microbiol.* 9 1295–1297. 10.2217/fmb.14.92 25517895

[B36] Koo H. Falsetta M. Klein M. (2013). The exopolysaccharide matrix: a virulence determinant of cariogenic biofilm. *J. Dent. Res.* 92 1065–1073. 10.1177/0022034513504218 24045647 PMC3834652

[B37] Krom B. P. Kidwai S. Ten Cate J. M. (2014). *Candida* and other fungal species: forgotten players of healthy oral microbiota. *J. Dent. Res.* 93 445–451. 10.1177/0022034514521814 24487378

[B38] Lee J. A. Robbins N. Xie J. L. Ketela T. Cowen L. E. (2016). Functional genomic analysis of *Candida albicans* adherence reveals a key role for the Arp2/3 complex in cell wall remodelling and biofilm formation. *PLoS Genet.* 12:e1006452. 10.1371/journal.pgen.1006452 27870871 PMC5147769

[B39] Lemos J. A. Quivey R. G. Koo H. Abranches J. (2013). *Streptococcus mutans*: a new gram-positive paradigm? *Microbiology* 159 436–445. 10.1099/mic.0.066134-0 23393147 PMC4083656

[B40] Lobo C. I. V. Rinaldi T. B. Christiano C. M. S. De Sales Leite L. Barbugli P. A. Klein M. I. (2019). Dual-species biofilms of *Streptococcus mutans* and *Candida albicans* exhibit more biomass and are mutually beneficial compared with single-species biofilms. *J. Oral. Microbiol.* 11:1581520. 10.1080/20002297.2019.1581520 31681463 PMC6807867

[B41] Metwalli K. H. Khan S. A. Krom B. P. Jabra-Rizk M. A. (2013). *Streptococcus mutans*, *Candida albicans* and the human mouth: a sticky situation. *PLoS Pathog.* 9:e1003616. 10.1371/journal.ppat.1003616 24146611 PMC3798555

[B42] Mitchell K. F. Zarnowskia R. Sancheza H. Edwarda J. A. Reinickea E. L. Netta J. E. (2015). Community participation in biofilm matrix assembly and function. *Proc. Nat. Acad. Sci. U.S.A.* 112 4092–4097. 10.1073/pnas.1421437112 25770218 PMC4386410

[B43] Montelongo-Jauregui D. Lopez-Ribot J. (2018). *Candida* interactions with the oral bacterial microbiota. *J. Fungi* 4:122. 10.3390/jof4040122 30400279 PMC6308928

[B44] Montelongo-Jauregui D. Saville S. P. Lopez-Ribot J. L. (2019). Contributions of *Candida albicans* dimorphism, adhesive interactions, and extracellular matrix to the formation of dual-species biofilms with *Streptococcus gordonii*. *mBio* 10:e01179-19. 10.1128/mBio.01179-01119 31213561 PMC6581863

[B45] Munro C. A. Bates S. Buurman E. T. Hughes H. B. MacCallum D. M. Bertram G. (2005). Mnt1p and Mnt2p of *Candida albicans* are partially redundant α-1, 2-mannosyltransferases that participate in O-linked mannosylation and are required for adhesion and virulence. *J. Biol. Chem.* 280 1051–1060. 10.1074/jbc.m411413200 15519997 PMC3749086

[B46] Nett J. Andes D. (2006). *Candida albicans* biofilm development, modeling a host-pathogen interaction. *Curr. Opin. Microbiol.* 9 340–345. 10.1016/j.mib.2006.06.007 16815078

[B47] Nett J. Lincoln L. Marchillo K. Massey R. Holoyda K. Hoff B. (2007). Putative role of beta-1,3 glucans in *Candida albicans* biofilm resistance. *Antimicrob. Agents Chemother.* 51 510–520. 10.1128/aac.01056-06 17130296 PMC1797745

[B48] Nett J. E. Sanchez H. Andes D. R. (2011). Interface of *Candida albicans* biofilm matrix-associated drug resistance and cell wall integrity regulation. *Eukaryot. Cell* 10 1660–1669. 10.1128/EC.05126-11 21666076 PMC3232725

[B49] Pereira D. Seneviratne C. Koga−Ito C. Samaranayake L. (2018). Is the oral fungal pathogen *Candida albicans* a cariogen? *Oral Dis.* 24 518–526. 10.1111/odi.12691 28513096

[B50] Pierce C. G. Vila T. Romo J. A. Montelongo-Jauregui D. Wall G. Ramasubramanian A. (2017). The *Candida albicans* biofilm matrix: Composition, structure and function. *J. Fungi (Basel)* 3:14. 10.3390/jof3010014 28516088 PMC5431293

[B51] Raja M. Hannan A. Ali K. (2010). Association of oral candidal carriage with dental caries in children. *Caries Res.* 44 272–276. 10.1159/000314675 20516688

[B52] Ramage G. Saville S. P. Thomas D. P. Lopez-Ribot J. L. (2005). *Candida* biofilms: an update. *Eukaryot. Cell* 4 633–638. 15821123 10.1128/EC.4.4.633-638.2005 PMC1087806

[B53] Rickard A. H. Gilbert P. High N. J. Kolenbrander P. E. Handley P. S. (2003). Bacterial coaggregation: an integral process in the development of multi-species biofilms. *Trends Microbiol.* 11 94–100. 10.1016/s0966-842x(02)00034-3 12598132

[B54] Ricker A. Vickerman M. Dongari-Bagtzoglou A. (2014). *Streptococcus gordonii* glucosyltransferase promotes biofilm interactions with *Candida albicans*. *J. Oral. Microbiol.* 6. 10.3402/jom.v6.23419 24490004 PMC3907680

[B55] Rouabhia M. Chmielewski W. (2012). Diseases anssociated with oral polymicrobial biofilms. *Open Mycol. J.* 6 27–32. 10.2174/1874437001206010027

[B56] Sampaio A. A. Souza S. E. Ricomini-Filho A. P. Del Bel Cury A. A. Cavalcanti Y. W. Cury J. A. (2019). *Candida albicans* increases dentine demineralization provoked by *Streptococcus mutans* biofilm. *Caries Res.* 53 322–331. 10.1159/000494033 30448846

[B57] Solis N. V. Filler S. G. (2012). Mouse model of oropharyngeal candidiasis. *Nat. Prot.* 7 637–642. 10.1038/nprot.2012.011 22402633 PMC3671943

[B58] Southard S. B. Specht C. A. Mishra C. Chen-Weiner J. Robbins P. W. (1999). Molecular analysis of the *Candida albicans* homolog of *Saccharomyces cerevisiae* MNN9, required for glycosylation of wall mannoproteins. *J. Bacteriol.* 181 7439–7448. 10.1128/jb.181.24.7439-7448.1999 10601199 PMC94199

[B59] Sultan A. S. Kong E. F. Rizk A. M. Jabra-Rizk M. A. (2018). The oral microbiome: a lesson in co-existence. *PLoS Pathog.* 14:e1006719. 10.1371/journal.ppat.1006719 29370304 PMC5784999

[B60] Sztajer H. Szafranski S. P. Tomasch J. Reck M. Nimtz M. Rohde M. (2014). Cross-feeding and interkingdom communication in dual-species biofilms of *Streptococcus mutans* and *Candida albicans*. *ISME J.* 8:2256. 10.1038/ismej.2014.73 24824668 PMC4992082

[B61] Tanner A. Kressirer C. Rothmiller S. Johansson I. Charlmers N. (2018). The caries microbiome: implications for reversing dysbiosis. *Adv. Dent. Res.* 29 78–85. 10.1177/0022034517736496 29355414

[B62] Valm A. M. (2019). The structure of dental plaque microbial communities in the transition from health to dental caries and periodontal disease. *J. Mol. Biol.* 431 2956–2969. 10.1016/j.jmb.2019.05.016 31103772 PMC6646062

[B63] Vu B. Chen M. Crawford R. Ivanova E. (2009). Bacterial extracellular polysaccharides involved in biofilm formation. *Molecules* 14 2535–2554. 10.3390/molecules14072535 19633622 PMC6254922

[B64] Wade W. G. (2013). The oral microbiome in health and disease. *Pharmacol. Res.* 69 137–143. 10.1016/j.phrs.2012.11.006 23201354

[B65] Wall G. Montelongo-Jauregui D. Vidal Bonifacio B. Lopez-Ribot J. L. Uppuluri P. (2019). *Candida albicans* biofilm growth and dispersal: contributions to pathogenesis. *Curr. Opin. Microbiol.* 11 1–6. 10.1016/j.mib.2019.04.001 31085405 PMC6842673

[B66] Williams D. Lewis M. (2011). Pathogenesis and treatment of oral candidosis. *J. Oral. Microbiol.* 3. 10.3402/jom.v3i0.5771 21547018 PMC3087208

[B67] Xiao J. Huang X. Alkhers N. Alzamil H. Alzoubi S. Wu T. T. (2018). *Candida albicans* and early childhood caries: a systematic review and meta-analysis. *Caries Res.* 52 102–112. 10.1159/000481833 29262404 PMC5828948

[B68] Xiao J. K. M. Falsetta M. L. Lu B. Delahunty C. M. Yates J. R. (2012). The exopolysaccharide matrix modulates the interaction between 3D architecture and virulence of a mixed-species oral biofilm. *PLoS Pathog.* 8:e1002623. 10.1371/journal.ppat.1002623 22496649 PMC3320608

[B69] Xu H. Dongari-Bagtzoglou A. (2015). Shaping the oral mycobiota: Interactions of opportunistic fungi with oral bacteria and the host. *Curr. Opin. Microbiol.* 27 65–70. 10.1016/j.mib.2015.06.002 26100661 PMC4577367

[B70] Xu H. Sobue T. Bertolini M. Thompson A. Dongari-Bagtzoglou A. (2016). *Streptococcus oralis* and *Candida albicans* synergistically activate mu-calpain to degrade E-cadherin from oral epithelial junctions. *J. infect. Dis.* 214 925–934. 10.1093/infdis/jiw201 27190184 PMC4996146

[B71] Xu H. Sobue T. Thompson A. Xie Z. Poon K. Ricker A. (2014). Streptococcal co-infection augments *Candida* pathogenicity by amplifying the mucosal inflammatory response. *Cell. Microbiol.* 16 214–231. 10.1111/cmi.12216 24079976 PMC3956708

[B72] Yang C. Scoffield J. Wu R. Deivanayagam C. Zou J. Wu H. (2018). Antigen I/II mediates interactions between *Streptococcus mutans* and *Candida albicans*. *Molec. Oral. Microbiol.* 33 283–291. 10.1111/omi.12223 29570954 PMC6041162

[B73] Zarnowski R. Sanchez H. Andes D. R. (2016). Large-scale production and isolation of *Candida* biofilm extracellular matrix. *Nat. Protoc.* 11 2320–2327. 10.1038/nprot.2016.132 27809313

[B74] Zarnowski R. Westler W. Lacmbouh G. Marita J. Bothe J. Bernhardt J. (2014). Novel entries in a fungal biofilm matrix encyclopedia. *mBio* 5:e01333-14. 10.1128/mBio.01333-14 25096878 PMC4128356

[B75] Zero D. T. Fontana M. Martinez-Mier E. A. Ferrera-Zandona A. Ando M. Gonzalez-Cabezas C. (2009). The biology, prevention, diagnosis and treatment of dental caries. *J. Am. Dent. Assoc.* 140(Suppl. 1) 25S–34S. 19723928 10.14219/jada.archive.2009.0355

