# Supplementary figures and images for: The Role of Candida albicans Secreted Polysaccharides in Augmenting Streptococcus mutans Adherence and Mixed Biofilm Formation: In vitro and in vivo Studies
Source: Front Microbiol. 2020 Feb 28;11:307. doi: 10.3389/fmicb.2020.00307 (PMC7093027; doi:10.3389/fmicb.2020.00307)

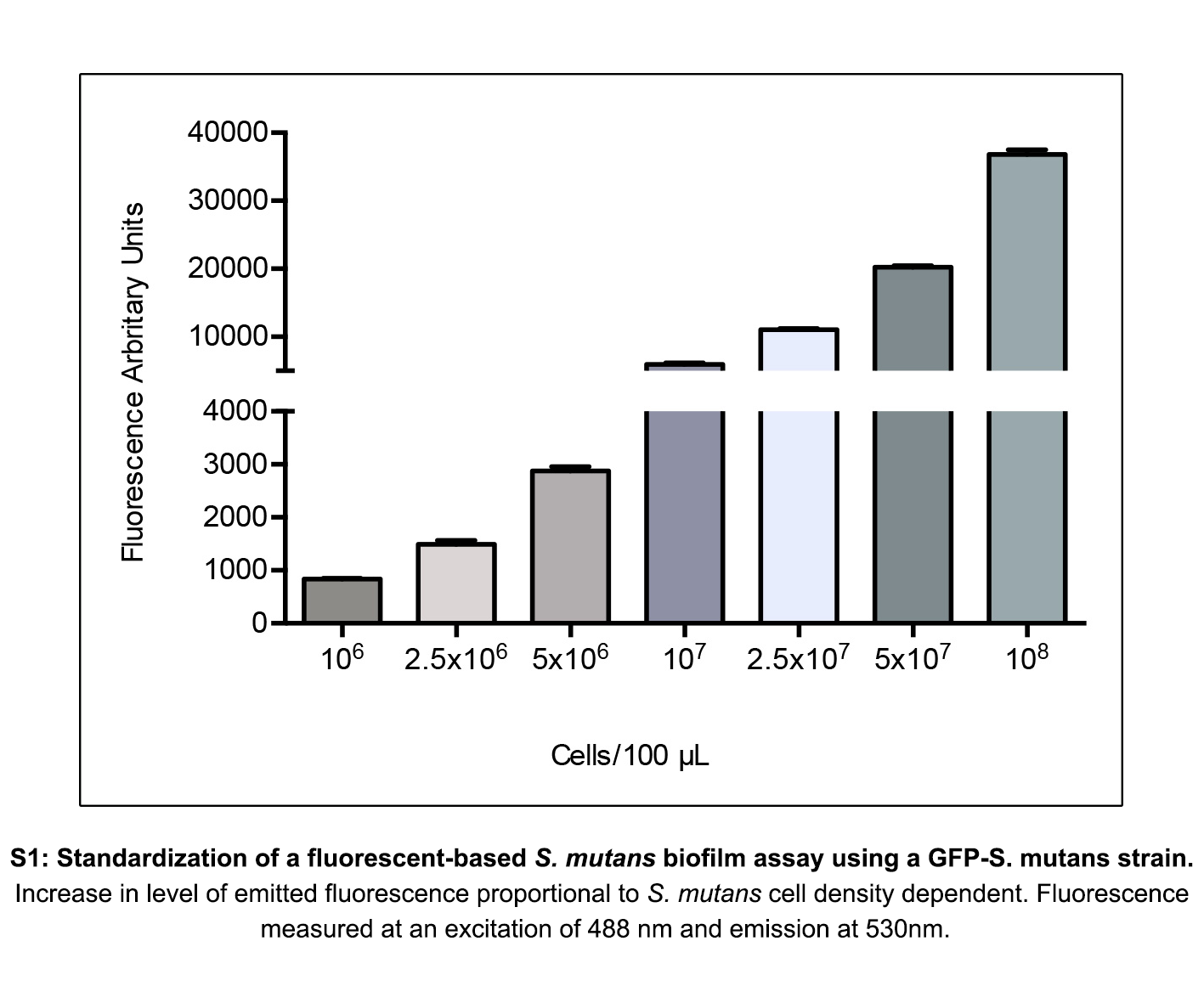

Supplement: Supplementary file 1 [file Image_1.TIF]
